# Asymmetric Optic Disc Edema in Astronauts: A Narrative Review Proposing an Interplay Between Ocular Venous Congestion and Glymphatic Transport

**DOI:** 10.3390/life16050831

**Published:** 2026-05-18

**Authors:** Peter Wostyn, Maiken Nedergaard, C. Robert Gibson, Thomas H. Mader

**Affiliations:** 1 Department of Psychiatry, PC Sint-Amandus, 8730 Beernem, Belgium; 2 Center for Translational Neuromedicine, Faculty of Health and Medical Sciences, University of Copenhagen, 2200 Copenhagen, Denmark; 3 Center for Translational Neuromedicine, University of Rochester Medical Center, Rochester, NY 14642, USA; 4 NASA Space Medicine Operations Division, Houston, TX 77058, USA; 5 KBR, Houston, TX 77002, USA; 6 NASA Ophthalmology Consultant, Moab, UT 84532, USA; tmader84@gmail.com

**Keywords:** astronaut, ocular glymphatic transport, ocular venous congestion, optic disc edema, spaceflight associated neuro-ocular syndrome, transverse sinus dominance

## Abstract

Spaceflight associated neuro-ocular syndrome (SANS) is a significant ophthalmic complication observed in astronauts during and after long-duration missions, characterized by optic disc edema, globe flattening, choroidal folds, and hyperopic shifts. Unlike papilledema in terrestrial idiopathic intracranial hypertension, optic disc edema in SANS is often asymmetric. The mechanisms underlying this asymmetry remain poorly understood. In this narrative review, we synthesize and critically interpret existing clinical observations, anatomical studies, neuroimaging findings, and experimental evidence, and propose that uneven ocular venous congestion, arising from microgravity-induced cephalad fluid shifts, pre-existing transverse sinus asymmetry, and orbital venous overload, leads to asymmetric optic disc edema by differentially disrupting anterograde ocular glymphatic transport between the eyes. This mechanistic framework highlights the interplay between venous hemodynamics and ocular glymphatic flow as a key factor in SANS pathophysiology. Targeted in-flight monitoring and ground-based analog studies will be essential to rigorously test this hypothesis. To this end, we outline a feasible experimental approach that prospectively integrates preflight cerebral magnetic resonance venography, providing data on transverse sinus dominance, with serial in-flight ophthalmic imaging on the International Space Station. This combined strategy could directly determine whether dural venous sinus anatomy predisposes to uneven ocular venous congestion and asymmetric optic disc edema in microgravity. Insights gained from this work may guide the development of effective countermeasures against SANS and broaden our understanding of ocular fluid dynamics under conditions of altered venous physiology on Earth.

## 1. Introduction

In 2011, Mader and colleagues first reported ophthalmic abnormalities in seven astronauts following long-duration space missions aboard the International Space Station (ISS) [1]. These findings included optic disc edema, globe flattening, choroidal folds, and hyperopic shifts in refraction [1]. The condition, initially termed ‘visual impairment and intracranial pressure syndrome’, was later renamed ‘spaceflight associated neuro-ocular syndrome’ (SANS) to reflect uncertainties regarding its underlying pathophysiology and the unclear role of intracranial pressure (ICP) [1]. Despite extensive investigation, the mechanisms driving SANS remain incompletely understood. The National Aeronautics and Space Administration (NASA) now recognizes SANS as one of the highest-priority health risks for human spaceflight, particularly as preparations advance for extended interplanetary missions, including human exploration of Mars. Among the diverse manifestations of SANS, asymmetric optic disc edema remains an unresolved and clinically significant feature [1]. Understanding the basis of this asymmetry is crucial for unraveling the underlying pathophysiology of SANS.

In this narrative review, we synthesize and critically interpret clinical observations, anatomical studies, neuroimaging findings, and experimental evidence to develop a coherent mechanistic framework addressing the asymmetry of optic disc edema observed in astronauts. The aim of this article is to integrate existing knowledge across disciplines in order to generate a unifying and testable pathophysiological hypothesis. Specifically, we propose that uneven ocular venous congestion, arising from the combined effects of microgravity-induced cephalad fluid shifts, pre-existing transverse sinus asymmetry, and orbital venous overload, leads to asymmetric optic disc edema by differentially disrupting anterograde ocular glymphatic transport between the eyes.

## 2. Materials and Methods

This manuscript is a narrative review aimed at providing a comprehensive, critical, and integrative synthesis of the existing literature on SANS, with a particular focus on asymmetric optic disc edema and its potential underlying mechanisms. The purpose of this review is to identify current knowledge gaps, propose a unifying mechanistic framework, and highlight directions for future research.

A structured but non-systematic literature search was performed to identify relevant publications related to SANS, optic disc edema, ICP, ocular venous outflow, dural venous sinus anatomy, and ocular glymphatic transport. The search was conducted using the following electronic databases: PubMed/MEDLINE, Scopus, and Web of Science.

Searches were performed up to April 2026 using combinations of relevant keywords, including but not limited to: “spaceflight associated neuro-ocular syndrome”, “SANS”, “optic disc edema”, “papilledema”, “intracranial pressure”, “microgravity”, “ocular glymphatic system”, “venous congestion”, and “transverse sinus asymmetry.” In addition, relevant studies were identified through manual screening of reference lists of key articles, review papers, and selected primary studies considered conceptually important for the development of the proposed hypothesis.

Given the narrative nature of this review, no predefined inclusion or exclusion criteria, study quality assessment, or formal risk-of-bias analysis was applied. Study selection was guided by scientific relevance, methodological rigor, and contribution to the conceptual understanding of SANS pathophysiology. The synthesis of the literature was qualitative and interpretative in nature, with the aim of integrating evidence across disciplines into a coherent mechanistic framework.

This study does not constitute a systematic review and was not conducted in accordance with PRISMA guidelines. Therefore, no PRISMA checklist or flow diagram is provided. The review did not involve human participants, animal experimentation, or the collection of new experimental or clinical data, and ethical approval was not required.

## 3. Discussion

### 3.1. The Role of Intracranial Pressure in SANS

SANS was originally thought to result from increased ICP due to microgravity-induced cephalad fluid shifts, leading to venous stasis in the head and neck [1]. This stasis could cause cerebral venous congestion and impair cerebrospinal fluid (CSF) outflow into the venous system, both of which may contribute to elevated ICP.

Nevertheless, the role of increased ICP in astronauts is increasingly debated, and it remains uncertain whether the optic disc edema observed in SANS truly represents papilledema, that is, swelling of the optic disc directly caused by elevated ICP [1]. Lumbar punctures in four astronauts with optic disc edema revealed only borderline or mildly elevated opening pressures of 21, 22, 28, and 28.5 cm H_2_O performed 19, 66, 12, and 57 days post mission, respectively [1]. Given the very invasive nature of lumbar punctures, they have never been performed during actual spaceflight. Nevertheless, although SANS shares some features with terrestrial idiopathic intracranial hypertension (IIH), astronauts typically do not experience hallmark IIH symptoms such as chronic headaches, pulsatile tinnitus, or diplopia [1]. This discrepancy suggests that elevated ICP may not be the sole or even the primary driver of optic disc edema in astronauts, underscoring the need to explore alternative mechanisms.

Findings from a parabolic flight study involving brief exposure to microgravity further challenge the notion that ICP is pathologically elevated in zero gravity, showing that ICP decreases relative to supine values but remains higher than in the 90 deg seated upright posture on Earth, indicating that complete removal of gravity prevents the normal reduction in ICP when upright [2]. The authors proposed that the absence of diurnal, postural decreases in ICP relative to intraocular pressure (IOP) under microgravity conditions may produce a persistently lower pressure gradient at the posterior eye, potentially leading to optic remodeling. In that context, it is important to note that exposure to microgravity is associated with an initial increase in IOP, which returns to values comparable to those on Earth after a few days [3].

### 3.2. Asymmetric Optic Disc Edema in SANS: Comparison with Papilledema in IIH

Comparing optic disc edema in SANS with papilledema in IIH may offer important insights into the mechanisms driving optic disc swelling in astronauts. By terrestrial standards, the optic disc edema observed in astronauts is often relatively mild and detected only by optical coherence tomography (OCT) [4]. When SANS was first documented, prior to the use of OCT, some cases were thought to be unilateral. However, with the advent of OCT, based on our experience, it has become evident that SANS-associated optic disc edema is usually present in both eyes. Importantly, whereas most terrestrial IIH-related papilledema is bilateral and typically symmetric, optic disc edema in SANS often shows asymmetry between the eyes [1]. Early observations suggested that, among astronauts who develop asymmetric optic disc edema following long-duration spaceflight, the right eye was preferentially affected [5]. However, with the advent of OCT and the availability of larger sample sizes, our observations suggest that there is no longer clear evidence supporting right-eye dominance.

In addition to differences in the edema itself, the characteristics of cotton wool spots (CWS) in astronauts and in IIH suggest distinct underlying mechanisms, which may provide important clues to the driving factors behind optic disc edema in SANS. CWS, thought to represent retinal nerve fiber layer infarcts caused by focal occlusion of precapillary retinal arterioles, have been observed on or near the optic disc in cases of papilledema [1]. In astronauts, these lesions are typically seen not only in the peripapillary area but also along the retinal vascular arcades, and to date, all documented cases of CWS in astronauts have been unilateral, based on our observations.

### 3.3. Asymmetric Disruption of Anterograde Ocular Glymphatic Transport in SANS

**Figure 1:**
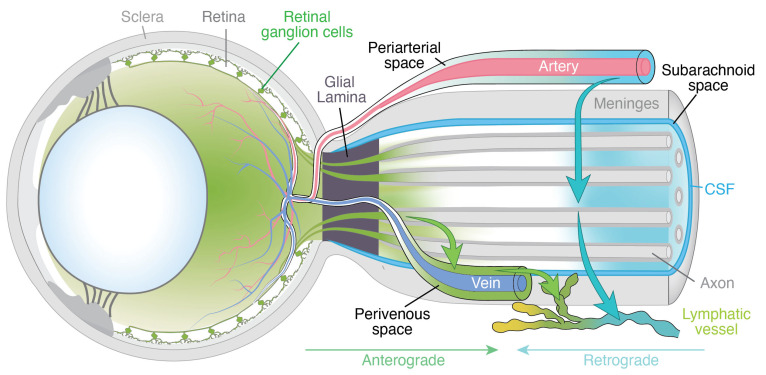
The ocular glymphatic system. Based on findings from rodent studies, the ocular glymphatic system supports bidirectional flow along the optic nerve, with anterograde transport from the retina and retrograde flow from the brain. The anterograde ocular glymphatic system facilitates the removal of fluid and metabolic waste from the posterior segment of the eye toward the optic nerve. Following intraocular tracer administration, molecules such as amyloid-β enter the retinal ganglion cells and perivenous spaces of the retina and optic nerve. Upon crossing the lamina barrier, amyloid-β is released from the axons of retinal ganglion cells, accumulating along the perivenous spaces of the optic nerve before being drained by the surrounding dural lymphatic vessels. Retrograde ocular glymphatic transport describes the flow of cerebrospinal fluid (CSF) into the optic nerve via periarterial spaces, from where CSF tracers subsequently drain through dural lymphatic vessels. Figure reproduced and modified with permission from [8].

We believe it is reasonable to interpret the asymmetry in SANS-associated optic disc edema in the context of altered and potentially asymmetric venous hemodynamics during spaceflight, which may asymmetrically disrupt anterograde ocular glymphatic transport. In 2020, Wang and colleagues [6] reported a study in rodents describing an ‘anterograde ocular glymphatic clearance pathway’ in addition to retrograde CSF transport along the periarterial spaces in the optic nerve (Figure 1). The anterograde pathway facilitates the removal of fluid and waste products from the intraocular space through retinal ganglion cell (RGC) axons and the perivenous spaces of the retina and optic nerve head. After crossing the lamina barrier, intra-axonal amyloid-β (Aβ) is transported along perivenous routes within the optic nerve and ultimately drained into dural lymphatic vessels (Figure 1). The authors showed that RGC axons use the hydrostatic pressure difference to facilitate fluid and solute movement across the optic nerve head [6,7]. More specifically, intra-axonal Aβ transport is driven by the trans-lamina cribrosa pressure difference, defined as the difference between IOP and ICP, with higher ICP suppressing this process [6,7]. While these findings provide important mechanistic insight, further studies are needed to determine whether, and how, a comparable glymphatic pathway operates in humans, given that rodents lack a true lamina cribrosa [6].

### 3.4. Ocular Venous Outflow and Microgravity Effects

**Figure 2:**
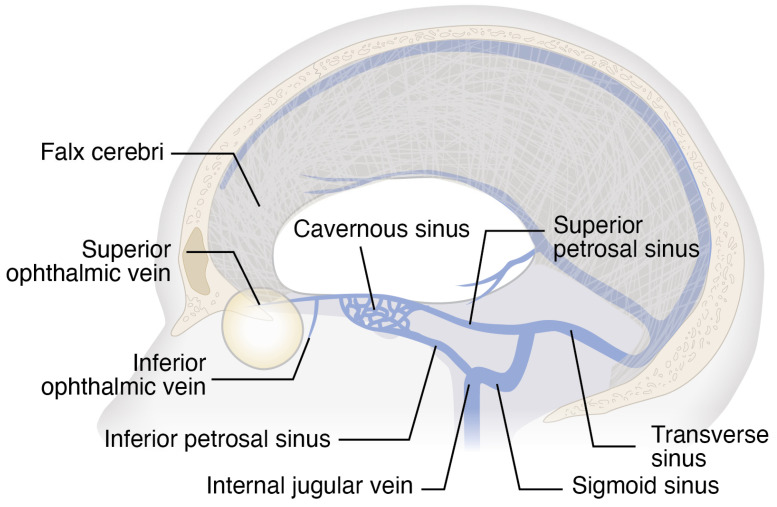
Orbital venous pathways connecting to dural venous sinuses. The superior and inferior ophthalmic veins drain into the cavernous sinus, which in turn communicates with the transverse sinus via the superior petrosal sinus, ultimately reaching the sigmoid sinus and internal jugular vein. The cavernous sinus can also drain directly into the internal jugular vein via the inferior petrosal sinus.

To better understand how asymmetric venous hemodynamics may lead to asymmetric optic disc edema in SANS, we will first briefly consider ocular venous outflow. Venous drainage occurs primarily via the central retinal vein and vortex veins. The central retinal vein exits the eye through the optic nerve head and drains either directly into the cavernous sinus or into the superior ophthalmic vein, which in turn empties into the cavernous sinus (Figure 1 and Figure 2) [9]. Vortex veins drain the choroid and empty into the superior and inferior ophthalmic veins [9], ultimately reaching the cavernous sinus. From the cavernous sinus, blood can flow via the superior petrosal sinus, which connects to the transverse sinus and ultimately leads to the sigmoid sinus before draining into the internal jugular vein (Figure 2). Additionally, the cavernous sinus can drain via the inferior petrosal sinus, which also empties into the internal jugular vein (Figure 2). Since ocular veins do not contain valves, which allows for retrograde flow, blood moving toward the head under microgravity may raise venous backpressure in both the retina and choroid.

A leading hypothesis proposes that SANS results from microgravity-induced cephalad fluid shifts, which cause venous stasis in the head and neck and subsequently lead to cerebral venous congestion [1]. This hypothesis is supported by a prospective cohort study by Marshall-Goebel and colleagues [10], who analyzed internal jugular vein flow in 11 ISS crew members participating in long-duration missions. The authors reported stagnant and even retrograde blood flow in the internal jugular veins of 6 astronauts, as well as internal jugular vein thrombosis in at least 1 crew member. Additionally, Rosenberg and colleagues [11] compared preflight and postflight dural venous sinus volumes in astronauts with and without SANS. They found that astronauts with SANS exhibited significantly greater increases in the volumes of the superior sagittal sinus and the right and left transverse/sigmoid sinuses than those without SANS. Based on these findings, they proposed that increased dural venous sinus compliance might predispose astronauts to SANS, while reduced compliance or relative rigidity in those without SANS could help protect against intracranial venous congestion. Taken together, their results suggest that astronauts with SANS experience greater intracranial venous congestion, which may contribute to the pathophysiology of the syndrome.

### 3.5. Anatomical Variation in Transverse Sinus Dominance

In addition to the observed increases in dural venous sinus volumes in astronauts with SANS, normal anatomic variation in transverse sinus dominance [12,13] may predispose certain individuals to asymmetric venous congestion. Magnetic resonance imaging (MRI) studies indicate that the transverse sinuses are highly asymmetric in the general population [12,13]. Ayanzen and colleagues [12] reviewed cerebral MR venograms from 100 individuals with normal MR imaging and found that the transverse sinus was right-dominant in 59% of cases, left-dominant in 25%, and codominant in 16%. Complementing these findings, Alper and colleagues [13] analyzed time-of-flight MR venograms from 105 individuals with normal MR studies. They reported that 20% of participants exhibited aplasia of the left transverse sinus, 39% had left sinus hypoplasia, 31% were symmetric, and hypoplasia or aplasia of the right transverse sinus was observed in only 6% and 4% of cases, respectively.

These observations underscore how variations in transverse sinus dominance could influence asymmetric venous pooling under microgravity conditions. Under microgravity-induced cephalad fluid shifts, this pre-existing asymmetry may lead to disproportionate venous pooling and elevated pressure within the dominant dural sinuses. The question, then, is whether this venous imbalance could also extend to the eye, manifesting as enhanced retinal vein dilation on the ipsilateral side. In this context, the question arises as to whether retinal vein dilation has indeed been observed during microgravity exposure. Recent findings by Binneboessel and colleagues [14] support this hypothesis, demonstrating that at least brief exposure to microgravity during parabolic flights induces significant retinal vein dilation. In addition, anecdotal observations indicate that the major retinal vessels, particularly the veins, tend to expand during long-duration spaceflight [15], further supporting the notion of ocular venous congestion under microgravity conditions. However, to date, no systematic in-flight investigations of retinal vascular changes during extended missions have yet been conducted, representing a critical gap in understanding the hemodynamic mechanisms underlying SANS. In the context of the present hypothesis, it would be highly informative to quantify how microgravity alters retinal vein diameter during long-duration spaceflight, establishing both the onset and temporal evolution of these changes. Such measurements can already be obtained with existing ISS ophthalmic imaging modalities, including retinal photography and OCT of the retina and optic nerve head.

Beyond the retinal circulation, it is also important to consider the choroid, a highly vascularized, sponge-like layer of the eye that may be particularly susceptible to microgravity-induced venous congestion [1]. A prospective cohort study by Macias and colleagues [16] demonstrated that long-duration spaceflight is associated with marked and sustained increases in peripapillary choroidal thickness. These changes were detectable early during the mission, persisted throughout microgravity exposure, and required 45 to 90 days after return to Earth to normalize. It has been hypothesized that microgravity-induced cephalad fluid shifts lead to venous congestion in the head and neck, resulting in elevated pressure within the vortex veins and impaired choroidal drainage [1]. This, in turn, may promote venous pooling and choroidal thickening [1].

Taken together, these observations support the hypothesis that dominance of the dural venous sinus may predispose the ipsilateral eye to increased venous pooling under microgravity conditions. A feasible and robust experimental approach to evaluate this possibility would involve prospectively integrating preflight cerebral MR venography datasets, providing detailed information on transverse sinus dominance, hypoplasia, and other venous asymmetries, with serial in-flight ophthalmic imaging. By correlating these preflight venous anatomical patterns with in-flight measurements of retinal vein diameter, peripapillary retinal nerve fiber layer thickness, peripapillary choroidal thickness, and optic nerve head morphology, it would be possible to directly test whether individuals with marked transverse sinus dominance exhibit preferential venous congestion and more pronounced optic disc changes in the ipsilateral eye during microgravity exposure.

### 3.6. Impact of Asymmetric Venous Congestion on Ocular Glymphatic Transport

Regarding the potential effects of altered and asymmetric venous hemodynamics during long-duration spaceflight on anterograde ocular glymphatic transport, it is important to distinguish between the intra-axonal and perivenous efflux routes, as these may rely on distinct biological mechanisms. In SANS, the development of optic disc edema may result from the combined effects of mild elevations in ICP and ocular venous congestion, with the latter potentially amplifying the impact of slight ICP increases on anterograde ocular glymphatic transport. Although slight increases in ICP may contribute to the condition to some extent, findings from the parabolic flight study reporting retinal vein dilation in participants [14], together with anecdotal evidence that the major retinal veins tend to dilate during long-duration spaceflight [15], suggest that local venous stasis may act as an amplifying factor that, when superimposed on mild ICP elevation, contributes to the development of optic disc edema under microgravity exposure.

**Figure 3:**
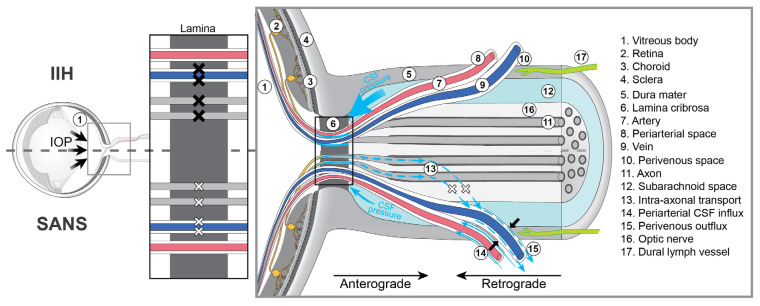
Schematic comparison of ocular glymphatic outflow alterations in SANS and IIH. The figure highlights key mechanistic differences between spaceflight associated neuro-ocular syndrome (SANS) and idiopathic intracranial hypertension (IIH), particularly the degree and symmetry of obstruction of intra-axonal and perivenous outflow. In SANS, ocular venous congestion is proposed to impair perivenous glymphatic efflux across and beyond the lamina cribrosa, while intra-axonal transport along retinal ganglion cell axons may be partially obstructed due to mildly elevated intracranial pressure (ICP), leading to mild optic disc edema. Dominance of the dural venous sinus may lead to asymmetric ocular venous congestion, potentially explaining the frequently observed asymmetry of optic disc edema in SANS. In IIH, markedly increased ICP causes a uniform and complete obstruction of both intra-axonal and perivenous outflow, resulting in bilateral, symmetric, and more pronounced papilledema. To illustrate these differences, the inset at the level of the lamina cribrosa uses lighter crosses to indicate partial obstruction of intra-axonal and perivenous outflow (as in SANS) and darker crosses to denote complete obstruction (as in IIH). CSF, cerebrospinal fluid; IOP, intraocular pressure. Figure reproduced and modified from [18]. Used under the Creative Commons Attribution (CC BY) 4.0 license.

If confirmed by systematic investigation during long-duration missions, such venous stasis, potentially amplified by the high compliance of the dural or orbital venous system, could lead to narrowing of the perivenous spaces, thereby increasing downstream resistance to perivenous outflow across and beyond the lamina cribrosa and consequently impeding glymphatic efflux along this route (Figure 3). In addition, mild elevations in ICP could hinder intra-axonal efflux via RGC axons, while the rise in perivenous downstream resistance beyond the lamina cribrosa could further impede this intra-axonal efflux, as tracer movement in the anterior optic nerve predominantly follows perivenous trajectories (Figure 3).

While intra-axonal transport may be reduced in SANS, a slight increase in ICP would not necessarily result in a complete blockade. Rather, it could cause a partial impairment which, together with impeded perivenous outflow, may contribute to the characteristic mild optic disc edema observed in astronauts. Because these hemodynamic and glymphatic changes are expected to occur bilaterally, this mechanism may also account for the fact that SANS-associated optic disc edema is usually present in both eyes. If venous volume and pressure overload within the dominant dural sinuses predispose to disproportionate venous congestion in the ipsilateral eye, this mechanism could plausibly explain the frequently observed asymmetry of optic disc edema in astronauts. By contrast, in IIH, markedly elevated ICP typically affects both optic nerves uniformly, potentially leading to a complete blockade of both intra-axonal and perivenous efflux across the lamina cribrosa (Figure 3), leading to bilateral, symmetric, and more pronounced papilledema compared with the generally milder optic disc edema observed in SANS.

Importantly, recent work by Sibony and colleagues [17] has demonstrated that patterns of ocular deformation in SANS differ fundamentally from those observed in IIH. The authors reported that in IIH patients with papilledema, the Bruch’s membrane opening (BMO) was displaced predominantly anteriorly toward the vitreous in 80% of eyes. In contrast, BMO displacement in crewmembers with SANS exhibited a bidirectional pattern, with 48% of eyes showing posterior displacement, 36% anterior displacement, and 16% no meaningful displacement. These findings indicate that SANS cannot be explained by elevated ICP alone and point to fundamental differences in the underlying biomechanics of the two conditions [17]. Sibony and colleagues [17] further proposed that the posterior ocular deformations observed in SANS may arise from a combination of factors acting individually or in concert, including a peripapillary indentation load related to choroidal expansion or orbital tissue pressure, interstitial prelaminar optic disc edema, alterations in the compliance of load-bearing structures, and differential displacement between the reference points and the BMO. Within the context of the present hypothesis, such changes may reflect the combined effects of ocular venous congestion and impaired glymphatic outflow.

### 3.7. Cotton Wool Spots and Venous Congestion

The observed distribution of CWS in astronauts can also be understood within the proposed framework of asymmetric ocular venous congestion. As noted above, astronauts typically develop CWS unilaterally, in the peripapillary region and along the retinal vascular arcades. This pattern aligns more closely with uneven ocular venous congestion than with uniform ICP elevation. Under microgravity, asymmetric ocular venous stasis may cause localized capillary outflow obstruction in areas directly affected by venous congestion. Such obstruction can lead to ischemia of the retinal nerve fiber layer, particularly within regions characterized by prominent venous drainage pathways, such as the peripapillary area and along the retinal vascular arcades, which are especially susceptible to elevated venous pressure and reduced perfusion pressure. When venous pressure rises, the outflow capacity of the major venous trunks diminishes, leading to increased capillary hydrostatic pressure and a reduction in perfusion pressure within their immediate drainage zones, thereby predisposing these areas to localized ischemia and CWS formation. In addition, venous distention may lead to narrowing of the perivenous spaces within the optic nerve head and retina, potentially impairing glymphatic clearance of neurotoxic waste products from the eye. Such glymphatic dysfunction may further contribute to the development of CWS, which can occur not only on or near the optic disc but also at more peripheral retinal locations. These mechanisms plausibly account for both the unilateral presentation and the characteristic peripapillary and vascular arcade distribution of CWS in astronauts. Within this interpretation, CWS provide further clinical evidence that disturbed ocular venous hemodynamics is a central driver of ocular pathology in SANS. Regarding CWS, other mechanisms may also contribute to their development. While CWS are well documented in association with papilledema in terrestrial IIH, they have also been observed following radiation exposure on Earth. Consequently, given the elevated radiation levels encountered during spaceflight, radiation may plausibly act as an additional contributing factor to their formation in astronauts [19].

### 3.8. Additional Mechanisms Contributing to Optic Disc Edema and Its Asymmetry

In addition to disturbed venous hemodynamics and impaired posterior fluid outflow from the eye, other mechanisms may also contribute to the development of optic disc edema in SANS. Increased capillary filtration at the optic nerve head [16], transudation of fluid from the choroidal vasculature that reaches the optic nerve head via the border tissue of Elschnig [8], and entry of excess CSF substrate into the eye [20] may further promote fluid stasis in the prelaminar region and thereby contribute to optic disc swelling in astronauts, along with other possible contributing mechanisms.

An alternative hypothesis proposes that localized compartmentalization of CSF within the orbital subarachnoid space (SAS) could represent an additional mechanism underlying optic disc edema in astronauts and may also help explain its asymmetric manifestation [1]. This hypothesis suggests that the unique cul-de-sac anatomy of the tightly confined, densely septated perioptic SAS is particularly vulnerable to disturbed fluid dynamics under prolonged microgravity. Coupled with the resulting cephalad fluid shifts, which can impede CSF absorption within the orbit via venous and lymphatic pathways, this vulnerability may lead to CSF sequestration and locally elevated optic nerve sheath pressures [1]. Even subtle anatomical differences between the optic nerve sheaths could then cause unequal CSF outflow, resulting in asymmetric optic disc edema. With regard to the hypothesis outlined in the present paper, it is intriguing to propose that asymmetric orbital venous congestion during spaceflight, arising from venous volume and pressure overload in the dominant dural sinuses, may preferentially impede CSF absorption within the ipsilateral orbit via venous pathways. This, in turn, could elevate pressure in the perioptic SAS on the same side, which may further impair ocular glymphatic outflow and thereby contribute to the asymmetry of optic disc edema observed in astronauts.

## 4. Conclusions

In conclusion, SANS represents a distinctive neuro-ocular condition in which asymmetric optic disc edema remains an unresolved and clinically significant feature. The evidence reviewed here suggests that asymmetric ocular venous congestion, driven by cephalad fluid shifts, pre-existing transverse sinus asymmetry, and orbital venous overload, may underlie this manifestation by asymmetrically disrupting anterograde ocular glymphatic transport. Additional factors, such as subtle differences in optic nerve sheath anatomy, may further contribute to asymmetric optic disc edema in astronauts. Elucidating how these mechanisms interact to produce the characteristic features of SANS is critical, not only for safeguarding astronauts’ ocular health during future long-duration missions but also for advancing our understanding of ocular fluid dynamics under conditions of altered venous physiology on Earth. Targeted in-flight monitoring and ground-based analog studies will be essential to test our hypothesis and to develop effective countermeasures against SANS.

## Data Availability

No new data were created or analyzed in this study. Data sharing is not applicable to this article.

## References

[1] Lee A.G., Mader T.H., Gibson C.R., Tarver W. (2017). Space flight-associated neuro-ocular syndrome. JAMA Ophthalmol..

[2] Lawley J.S., Petersen L.G., Howden E.J., Sarma S., Cornwell W.K., Zhang R., Whitworth L.A., Williams M.A., Levine B.D. (2017). Effect of gravity and microgravity on intracranial pressure. J. Physiol..

[3] Masalkhi M., Ong J., Waisberg E., Berdahl J., Lee A.G. (2023). Intraocular pressure during spaceflight and risk of glaucomatous damage in prolonged microgravity. Encyclopedia.

[4] Valencia W.E., Mason S.S., Brunstetter T.J., Sargsyan A.E., Schaefer C.M., Tarver W.J., Van Baalen M.G., Gibson C.R., Lee A.G., Danilichev S.N. (2023). Evaluation of optic disc edema in long-duration spaceflight crewmembers using retinal photography. J. Neuroophthalmol..

[5] Mader T.H., Gibson C.R., Hart S.F., Lee A.G. (2016). Asymmetric papilledema in idiopathic intracranial hypertension: Comment. J. Neuroophthalmol..

[6] Wang X., Lou N., Eberhardt A., Yang Y., Kusk P., Xu Q., Förstera B., Peng S., Shi M., Ladrón-De-Guevara A. (2020). An ocular glymphatic clearance system removes β-amyloid from the rodent eye. Sci. Transl. Med..

[7] Rangroo Thrane V., Hynnekleiv L., Wang X., Thrane A.S., Krohn J., Nedergaard M. (2021). Twists and turns of ocular glymphatic clearance—New study reveals surprising findings in glaucoma. Acta Ophthalmol..

[8] Wang X., Delle C., Nedergaard M., Wostyn P. (2026). Glymphatic transport and ocular diseases. Prog. Retin. Eye Res..

[9] Mampre D., Spaide R., Mason S., Van Baalen M., Gibson C.R., Mader T.H., Wostyn P., Briggs J., Brown D., Lee A.G. (2025). Spaceflight associated neuro-ocular syndrome as a potential variant of venous overload choroidopathy. Aerosp. Med. Hum. Perform..

[10] Marshall-Goebel K., Laurie S.S., Alferova I.V., Arbeille P., Auñón-Chancellor S.M., Ebert D.J., Lee S.M.C., Macias B.R., Martin D.S., Pattarini J.M. (2019). Assessment of jugular venous blood flow stasis and thrombosis during spaceflight. JAMA Netw. Open.

[11] Rosenberg M.J., Coker M.A., Taylor J.A., Yazdani M., Matheus M.G., Blouin C.K., Al Kasab S., Collins H.R., Roberts D.R. (2021). Comparison of dural venous sinus volumes before and after flight in astronauts with and without spaceflight-associated neuro-ocular syndrome. JAMA Netw. Open.

[12] Ayanzen R.H., Bird C.R., Keller P.J., McCully F.J., Theobald M.R., Heiserman J.E. (2000). Cerebral MR venography: Normal anatomy and potential diagnostic pitfalls. AJNR Am. J. Neuroradiol..

[13] Alper F., Kantarci M., Dane S., Gumustekin K., Onbas O., Durur I. (2004). Importance of anatomical asymmetries of transverse sinuses: An MR venographic study. Cerebrovasc. Dis..

[14] Binneboessel S., Gerdes N., Baertschi M., Kaya S., Geerling G., Kelm M., Jung C. (2024). Changes in ocular perfusion and pressure changes in gravitational alteration contribute to spaceflight-associated neuro-ocular syndrome. Arterioscler. Thromb. Vasc. Biol..

[15] Brunstetter T. (2025). Personal communication.

[16] Macias B.R., Patel N.B., Gibson C.R., Samuels B.C., Laurie S.S., Otto C., Ferguson C.R., Lee S.M.C., Ploutz-Snyder R., Kramer L.A. (2020). Association of long-duration spaceflight with anterior and posterior ocular structure changes in astronauts and their recovery. JAMA Ophthalmol..

[17] Sibony P.A., Laurie S.S., Ferguson C.R., Pardon L.P., Young M., Rohlf F.J., Macias B.R. (2023). Ocular deformations in spaceflight-associated neuro-ocular syndrome and idiopathic intracranial hypertension. Investig. Ophthalmol. Vis. Sci..

[18] Wostyn P., Nedergaard M. (2025). Could sleep be an antidote to optic disc edema in astronauts?. Life.

[19] Waisberg E., Ong J., Paladugu P., Kamran S.A., Zaman N., Tavakkoli A., Lee A.G. (2024). Radiation-induced ophthalmic risks of long duration spaceflight: Current investigations and interventions. Eur. J. Ophthalmol..

[20] Wostyn P., Mader T.H., Gibson C.R., Nedergaard M. (2025). New insights in brain-to-eye transport: Can excess cerebrospinal fluid in astronauts escape into the eye?. Eye.

